# Efficacy and safety of tenofovir and entecavir in patients with chronic hepatitis B-related cirrhosis: a systematic review and meta-analysis

**DOI:** 10.3389/fphar.2025.1507117

**Published:** 2025-01-20

**Authors:** Lu Cao, Li Li, Lixing Yang, Nan Zhou, Yu Zhang

**Affiliations:** ^1^ Department of Pharmacy, Shaanxi Provincial People’s Hospital, Xi’an, Shaanxi, China; ^2^ Hepatobiliary Surgery, Shaanxi Provincial People’s Hospital, Xi’an, Shaanxi, China

**Keywords:** meta-analysis, tenofovir, entecavir, chronic hepatitis B, cirrhosis

## Abstract

**Objectives:**

To systematically evaluate the efficacy and safety of tenofovir and entecavir in chronic hepatitis B-related cirrhosis.

**Methods:**

A comprehensive search was conducted in databases including PubMed, Web of Science, Embase and Cochrane Library from the inception until June 2024. Studies on the use of tenofovir and entecavir for chronic hepatitis B-related cirrhosis were collected.

**Results:**

A total of 14 studies involving 14,208 patients were included. The meta-analysis revealed that tenofovir significantly reduced the cumulative incidence of hepatocellular carcinoma and cumulative mortality compared to entecavir in East Asian popupation, while in non East Asian populations, the two groups are roughly equivalent. After 48 weeks, the hepatitis B virus-deoxyribonucleic acid clearance rate in the tenofovir group were comparable to the entecavir group. Both tenofovir and entecavir showed similar effect in reducing the incidence of hepatic encephalopathy. Compared with the entecavir group, patients in the tenofovir group, including tenofovir disoproxil fumarate and tenofovir alafenamide fumarate showed a significant increase in estimated glomerular filtration rate after 48 weeks of treatment.

**Conclusion:**

Compared to entecavir, tenofovir significantly reduced the cumulative incidence of hepatocellular carcinoma and cumulative mortality in chronic hepatitis B-related cirrhosis in East Asian population. However, both drugs were comparable in terms of hepatitis B virus-deoxyribonucleic acid clearance and hepatic encephalopathy. Tenofovir did not significantly cause renal dysfunction, but instead improved estimated glomerular filtration rate levels compared with entecavir. Randomized controlled trials with larger sample size are still needed for validation.

**Systematic Review Registration:**

https://www.crd.york.ac.uk/prospero/, identifier CRD42024588432.

## 1 Introduction

Chronic Hepatitis B Virus (HBV) infection represents a significant global public health concern, with an estimated 296 million individuals affected worldwide in 2019 (Hu et al., 2020; Liu et al., 2019; Chen et al., 2020; Huang et al., 2023; Lee et al., 2022; Lin et al., 2024). Chronic Hepatitis B (CHB) can lead to liver fibrosis, cirrhosis, and hepatocellular carcinoma (HCC), resulting in over 887,000 deaths annually (Wang et al., 2021). Cirrhosis is a late-stage manifestation of chronic liver disease, wherein patients progress from compensated to decompensated liver function, accompanied by complications such as gastrointestinal bleeding, hepatic encephalopathy, infections and HCC, all of which severely impact quality of life and prognosis (Yoshiji et al., 2021). Studies had reported that, in untreated decompensated CHB-related cirrhosis patients, the cumulative incidence of HCC was approximately 53.1% (Huang et al., 2023), with a 5-year survival rate ranging between 14% and 35% (Wang et al., 2021). However, the standardized use of antiviral therapy to suppress HBV replication and reduce viral load can effectively halt the progression of cirrhosis, and in some cases, even reverse it (Wang et al., 2021).

Nucleos(t)ide analogues (NAs), such as entecavir (ETV), tenofovir disoproxil fumarate (TDF), and tenofovir alafenamide fumarate (TAF), are recommended as first-line antiviral agents for CHB-related cirrhosis in both domestic and international guidelines due to their potent antiviral efficacy and low resistance rates, making them suitable for long-term therapy (Sarin et al., 2016; Terrault et al., 2018; Lampertico et al., 2017). Studies had demonstrated substantial efficacy in fibrosis and cirrhosis reversal with both tenofovir and entecavir in CHB-related cirrhosis patients (Patrick et al., 2015). However, conflicting conclusions had emerged regarding the long-term clinical outcomes of tenofovir and entecavir in these patients, particularly with respect to the 5-year HCC risk. For instance, a study by Huang et al. (2023) showed that tenofovir significantly reduced the risk of HCC in CHB-related cirrhosis patients, while more recent research by Lee et al. (2022) and Lin et al. (2024) reported that tenofovir and entecavir exhibited comparable effect in reducing HCC risk in decompensated CHB-related cirrhosis patients. Therefore, the relationship between entecavir and tenofovir in long-term clinical outcomes and the risk of complications in patients with CHB-related cirrhosis remains unclear.

In our study, Meta-analysis was used to compare the differences in liver cirrhosis related complications, mortality rate, and renal function impairment between entecavir and tenofovir in the treatment of CHB related cirrhosis to evaluate the efficacy and safety of entecavir and tenofovir in the treatment of patients with CHB-related cirrhosis, with a view to providing high-quality, evidence-based medical evidence for the clinical use.

## 2 Materials and methods

### 2.1 Inclusion criteria

(1) Participants: Patients aged 18 years or older diagnosed with CHB-related cirrhosis.
(2) Interventions/Comparisions: The intervention group received oral tenofovir monotherapy, including TAF or TDF, while the control group received oral entecavir monotherapy. The treatment duration was at least 6 months, and the dosage followed the drug instructions and guideline recommendation.
(3) Outcomes: Primary outcomes included the cumulative incidence of HCC and cumulative mortality. Secondary outcomes included HBV-deoxyribonucleic acid (HBV-DNA) clearance rate and incidence of hepatic encephalopathy. Safety outcome included renal dysfunction, assessed using the estimated glomerular filtration rate (eGFR).
(4) Study types: Published randomized controlled trial (RCTs) and retrospective studies.

### 2.2 Exclusion criteria

(1) Studies involving non-cirrhotic or non-CHB-related cirrhosis patients; (2) Patients co-infected with other hepatitis viruses (A, C, D, or E) or human immunodeficiency virus; (3) Patients with concurrent alcoholic liver disease, autoimmune liver disease, or drug-induced liver injury; (4) Patients receiving additional antiviral drugs or traditional Chinese medicine; (5) Patients with HCC or other malignancies; (6) Patients who had undergone liver transplantation; (7) Patients with contraindications to entecavir or tenofovir; (8) Patients with severe heart, brain, kidney, or hematologic diseases; (9) Pregnant or lactating patients; (10) Studies that did not assess the defined outcome measures; (11) Duplicate publications; (12) Case reports, reviews, or conference abstracts; (13) Publications in non-English language; (14) Studies for which the full text could not be obtained despite attempts to contact the authors.

### 2.3 Literature search strategy

A comprehensive search was conducted in PubMed, Web of Science, Embase, and Cochrane Library from their inception to June 2024. The search terms included: (“cirrhosis” OR “liver cirrhosis” OR “hepatic cirrhosis” OR “liver fibrosis”) AND (“tenofovir” OR “tenofovir disoproxil fumarate” OR “tenofovir alafenamide fumarate”) AND (“entecavir”). Both subject terms and free-text terms were used, with adjustments made for specific databases. The references of the included studies were also searched to retrieve additional relevant materials.

### 2.4 Data screening and extraction

Two researchers (L.C and N.Z) independently screened the literature and extracted the data, with cross-checking for accuracy. Discrepancies were resolved through discussion or by involving the third one. Extracted data including: (1) Basic information about the study (first author, publication date, country, and study design); (2) Clinical characteristics of participants (age, sample size, genders and diseases); (3) Interventions and conparisions; (4) Clinical outcomes; (5) Quality assessment indicators.

### 2.5 Quality assessment of included studies

The methodological quality of RCT studies was evaluated using the Risk of Bias 2 tool 2.0 (RoB 2.0) recommended by the Cochrane Handbook, which includes five modules: bias generated during randomization, bias deviating from established interventions, bias due to missing outcome data, bias due to outcome measurement, and bias due to selective reporting (Sterne et al., 2019). Retrospective studies were evaluated using the Newcastle-Ottawa Scale (NOS) (Stang, 2010), which assesses the representativeness of the study population, the comparability of study groups, the adequacy of follow-up, and the completeness of outcome reporting. Studies scoring between 5 and 9 points were considered to have a low risk of bias and were included in the meta-analysis, with higher scores indicating lower bias.

### 2.6 Statistical analysis

The statistical analysis was performed using Review Manager (RevMan) Version 5.0 (Copenhagen: The Nordic Cochrane Centre, The Cochrane Collaboration, 2008). For dichotomous outcomes, the odds ratio (OR) and its 95% confidence interval (CI) were calculated. For continuous outcomes, the weighted mean difference (WMD) and its 95% CI were used. Heterogeneity among the included studies was assessed using Cochran Q test: if P > 0.1 and I^2^ ≤ 50%, indicating no significant heterogeneity, a fixed-effects model was applied; otherwise, a random-effects model was used. Sensitivity analyses were conducted using Stata 15.0 software for indicators with a large number of included literature, a high degree of heterogeneity and analyzed using a random-effects model. Publication bias for outcomes with at least 8 studies incuded was assessed using Egger’s test (Cao L. et al., 2022). P < 0.05 was considered statistically significant.

## 3 Results

### 3.1 Literature search results and methodological quality assessment

**FIGURE 1:**
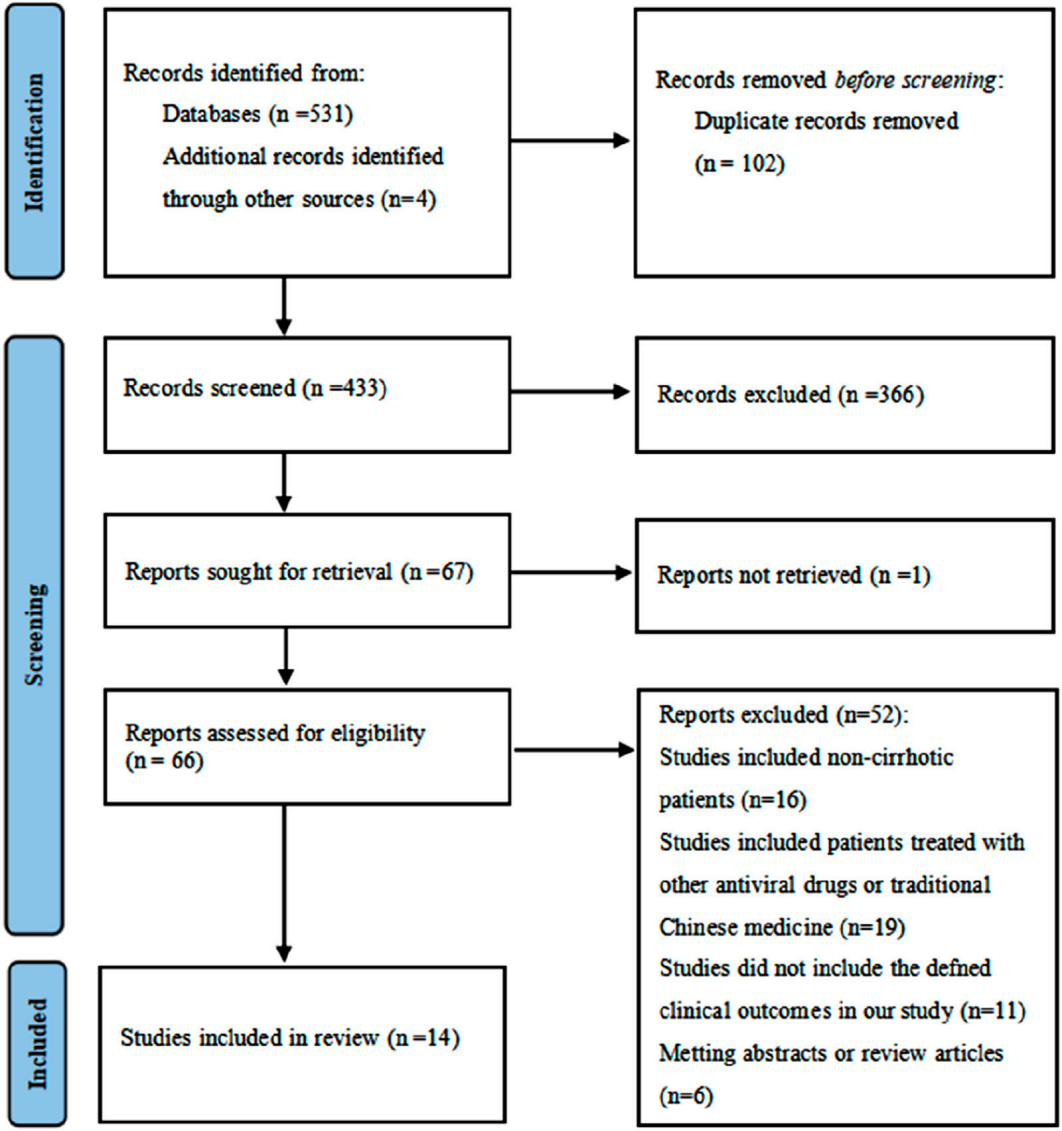
Literature screening flowchart.

A total of 535 studies were identified using the predefined search terms. After screening, 14 studies (Hu et al., 2020; Chen et al., 2020; Huang et al., 2023; Lee et al., 2022; Lin et al., 2024; Alkan et al., 2020; Cholongitas et al., 2015; Goyal et al., 2015; Gui et al., 2021; Huang et al., 2022; Köklü et al., 2013; Park et al., 2017; Tsai et al., 2017; Yao et al., 2021) were included in the systematic review and meta-analysis. The flowchart of the literature selection process was shown in Figure 1. All the 14 included studies were retrospective in nature, with NOS scores ranging from 6 to 9, which resulted in all studies being classified as high quality. The overall methodological quality was acceptable, with specific scores detailed in Table 1.

**TABLE 1:** Characteristics of included studies.

Author, Year	Country	Study design	Age/years	Gender (male/female)	Study population	Intervention/Control (sample size)		Outcome measures	NOS score
						tenofovir	entecavir		
Lin, 2024	China	Single-center retrospective cohort study	NA	205/81	Patients with CHB-related cirrhosis	TDF: 300 mg qd (n = 88)	ETV: 0.5 mg qd (n = 198)	Cumulative HCC incidence; Cumulative mortality	8
Huang, 2023	China	Multicenter retrospective study	>20	5,407/1909	Newly diagnosed patients with HBV-related cirrhosis	TDF: 300 mg qd (n = 3,658)	ETV: 0.5 mg qd (n = 3,658)	Cumulative HCC incidence; Cumulative mortality	9
Huang, 2022	China	Multicenter retrospective cohort study	≥18	1,049/404	HBV-related compensated cirrhosis	TDF: 300 mg qd (n = 188)	ETV: 0.5 mg qd (n = 1,265)	Cumulative HCC incidence; Cumulative mortality	9
Lee, 2022	Taiwan, China	Single-center retrospective cohort study	≥18	NA	Patients with decompensated CHB-related cirrhosis	TDF: 300 mg qd (n = 35)	ETV: 0.5 mg qd (n = 149)	Cumulative HCC incidence; Cumulative mortality	8
Gui, 2021	China	Single-center retrospective study	≥18	NA	Patients with compensated CHB-related cirrhosis	TDF: 300 mg qd (n = 105)	ETV: 0.5 mg qd (n = 937)	Cumulative HCC incidence	8
Yao, 2021	China	Multicenter retrospective cohort study	≥18	75/62	Patients with CHB-related cirrhosis and portal hypertension	TDF: 300 mg qd (n = 35) TAF: 25 mg qd (n = 32)	ETV: 0.5 mg qd (n = 70)	Cumulative HCC incidence; Cumulative mortality; Hepatic encephalopathy incidence; eGFR	7
Hu, 2020	China	Single-center retrospective cohort study	≥18	653/241	Patients with compensated CHB-related cirrhosis	TDF: 300 mg qd (n = 216)	ETV: 0.5 mg qd (n = 678)	Cumulative HCC incidence	9
Chen, 2020	China	Multicenterretretrospective study	≥18	1,149/411	Patients with CHB-related cirrhosis	TDF: 300 mg qd (n = 567)	ETV: 0.5 mg qd (n = 993)	Cumulative HCC incidence; HBV-DNA clearance	7
Alkan, 2020	Turkey	Single-center retrospective study	≥18	NA	Patients with CHB-related cirrhosis	TDF: 300 mg qd (n = 32)	ETV: 0.5 mg qd (n = 26)	HBV-DNA clearance	7
Park, 2017	Korea	Single-center retrospective cohort study	≥18	155/80	Treatment-naive patients with CHB-related cirrhosis	TDF: 300 mg qd (n = 73)	ETV: 0.5 mg qd (n = 162)	eGFR	6
Tsai, 2016	Taiwan, China	Single-center retrosepctive cohort study	≥18	322/120	Patients with CHb-related cirrhosis	TDF: 300 mg qd (n = 83)	ETV: 0.5 mg qd (n = 359)	HBV-DNA clearance	9
Cholongitas, 2015	Greece	Multicenter retrospective study	≥18	33/19	Patients with decompensated CHb-related cirrhosis	TDF: 300 mg qd (n = 31)	ETV: 0.5 mg qd (n = 21)	Cumulative HCC incidence; Cumulative mortality	8
Goyal, 2015	India	Multicenter retrospective cohort study	24–65	280/120	Patients with CHB-related cirrhosis ineligible for liver transplantation	TDF: 300 mg qd (n = 220)	ETV: 0.5 mg qd (n = 180)	Cumulative HCC incidence; Cumulative mortality; HBV-DNA clearance; Hepatic encephalopathy incidence	8
Koklu, 2013	Turkey	Single-center retrospective cohort study	≥18	114/35	Patients treated with any antiviral drug for more than 12 months after cirrhosis	TDF: 300 mg qd (n = 72)	ETV: 0.5 mg qd (n = 77)	Cumulative HCC incidence; Cumulative mortality; Hepatic encephalopathy incidence	8

Note: NA: not available; CHB: Chronic Hepatitis B; HCC: hepatocellular carcinoma; ETV: entecavir; TDF: tenofovir disoproxil fumarate; TAF: tenofovir alafenamide fumarate; eGFR: estimated glomerular filtration rate; HBV-DNA: HBV-deoxyribonucleic acid; NOS: Newcastle-Ottawa Scale.

### 3.2 Characteristics of included studies

A total of 14 studies, involving 14,208 patients, were included in the analysis. Of these, 5,435 patients were in the tenofovir group and 8,773 patients were in the entecavir group. The study designs of both the experimental and control groups took into account the basic characteristics and disease types of the patients, ensuring comparability between the two groups. The detailed characteristics of the included studies are presented in Table 1.

### 3.3 Meta-analysis results

#### 3.3.1 Cumulative HCC incidence

**FIGURE 2:**
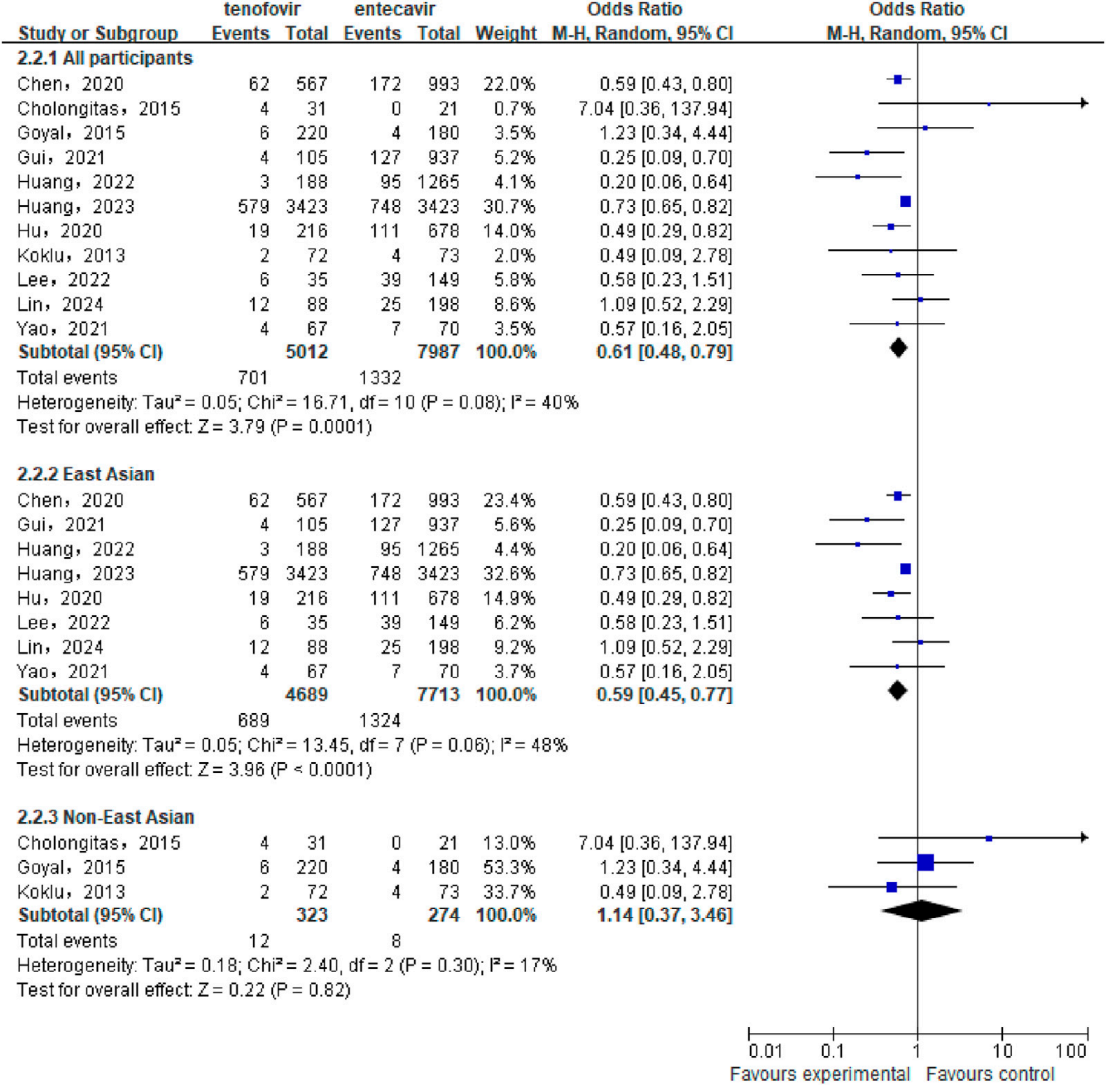
Cumulative HCC Incidence in the tenofovir and entecavir Groups.

A total of 11 studies (Hu et al., 2020; Chen et al., 2020; Huang et al., 2023; Lee et al., 2022; Lin et al., 2024; Cholongitas et al., 2015; Goyal et al., 2015; Gui et al., 2021; Huang et al., 2022; Köklü et al., 2013; Yao et al., 2021) involving 12,999 CHB-related cirrhosis patients were included to compare the cumulative incidence of HCC between the tenofovir and entecavir groups. Statistical heterogeneity was observed among the included studies (P = 0.08, I^2^ = 40%), so a random-effect model was used for the analysis. The meta-analysis results indicated that the cumulative incidence of HCC in the tenofovir group (13.99%) was significantly lower than in the entecavir group (16.68%) [OR: 0.61, 95% CI: 0.48–0.79, P = 0.0001], as shown in Figure 2. Subgroup analysis showed that in the East Asian population, the cumulative incidence of HCC in the tenofovir group (14.69%) was significantly lower than that in the entecavir group (17.17%) [OR: 0.59, 95% CI: 0.45–0.77, P < 0.0001], while in the non East Asian population, there was no significant difference in the cumulative incidence of HCC between the two groups [OR: 1.14, 95% CI: 0.37–3.46, P = 0.82].

#### 3.3.2 Cumulative mortality

**FIGURE 3:**
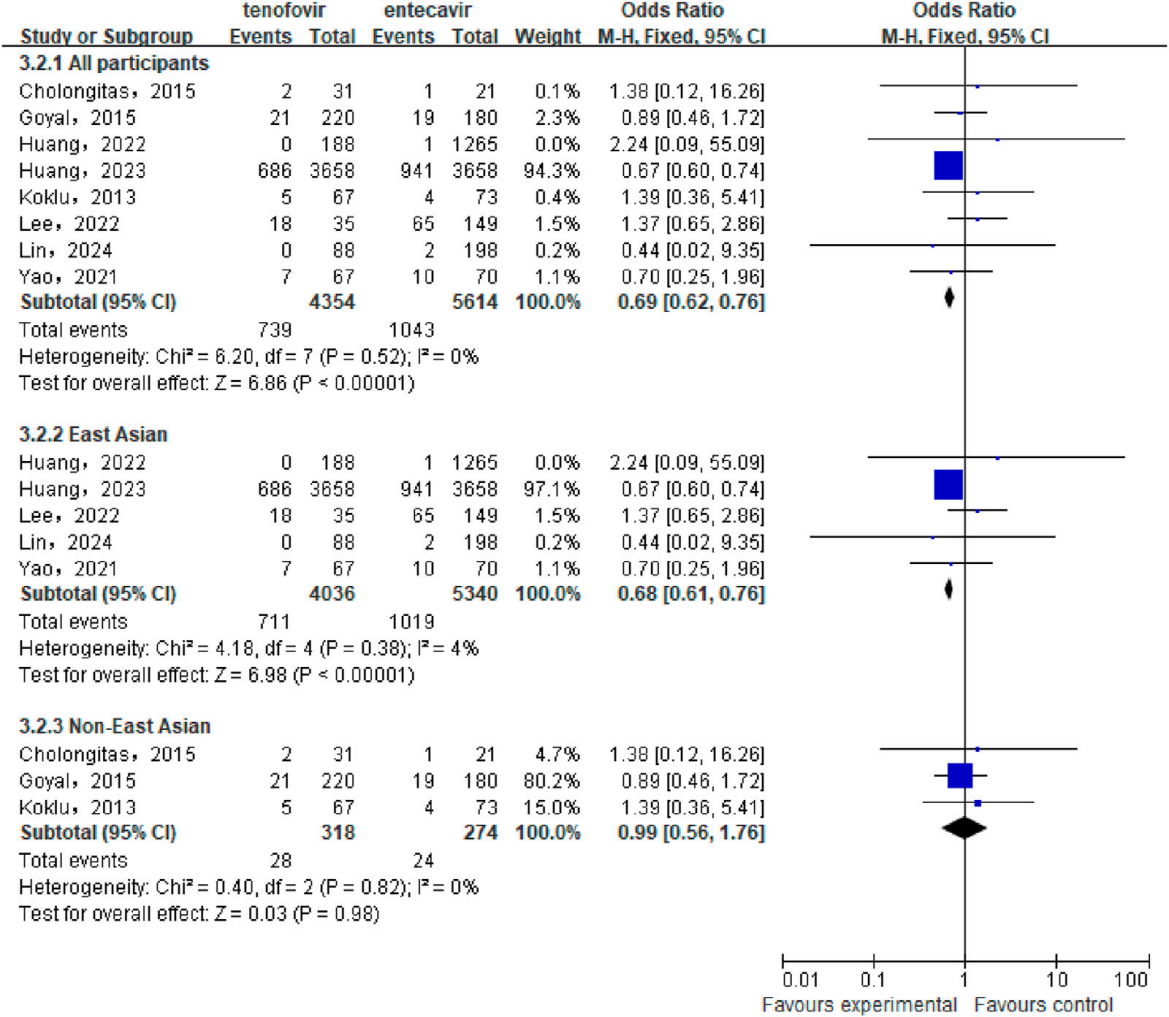
Cumulative Mortality in the tenofovir and entecavir Groups.

A total of 8 studies (Huang et al., 2023; Lee et al., 2022; Lin et al., 2024; Cholongitas et al., 2015; Goyal et al., 2015; Huang et al., 2022; Köklü et al., 2013; Yao et al., 2021) involving 9,968 patients were included to compare the cumulative mortality between the tenofovir and entecavir groups in CHB-related cirrhosis patients. No statistical heterogeneity was observed among the included studies (P = 0.52, I^2^ = 0%), so a fixed-effect model was used for the analysis. The meta-analysis results showed that the cumulative mortality in the tenofovir group (16.97%) was significantly lower than that in the entecavir group (18.58%), with a statistically significant difference between the two groups [OR: 0.69, 95% CI: 0.62–0.76, P < 0.00001], as shown in Figure 3. Subgroup analysis showed that in the East Asian population, the cumulative mortality in the tenofovir group (17.62%) was significantly lower than that in the entecavir group (19.08%) [OR: 0.68, 95% CI: 0.61–0.76, P < 0.00001], while in the non East Asian population, there was no significant difference in the cumulative mortality between the two groups [OR: 0.99, 95% CI: 0.56–1.76, P = 0.98].

#### 3.3.3 HBV-DNA clearance rate

**FIGURE 4:**
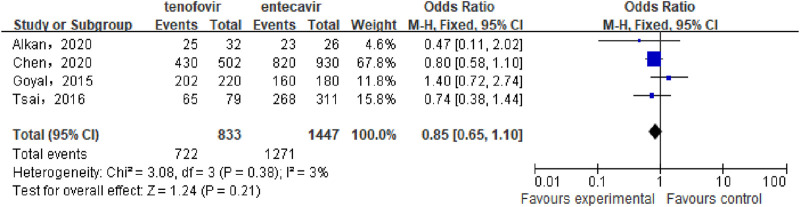
HBV-DNA Clearance Rate in the tenofovir and entecavir Groups after 48 weeks of Treatment.

A total of 4 studies (Chen et al., 2020; Alkan et al., 2020; Goyal et al., 2015; Tsai et al., 2017) involving 2,280 patients compared the HBV-DNA clearance rate between the tenofovir and entecavir groups in CHB-related cirrhosis patients after 48 weeks of treatment. No statistical heterogeneity was observed among the included studies (P = 0.38, I^2^ = 3%), so a fixed-effect model was used for the analysis. The results showed that after 48 weeks, the HBV-DNA clearance rate in the tenofovir group (86.67%) was similar to that in the entecavir group (87.84%), with no statistically significant difference between the two groups [OR: 0.85, 95% CI: 0.65–1.10, P = 0.21], as shown in Figure 4.

#### 3.3.4 Incidence of hepatic encephalopathy

**FIGURE 5:**
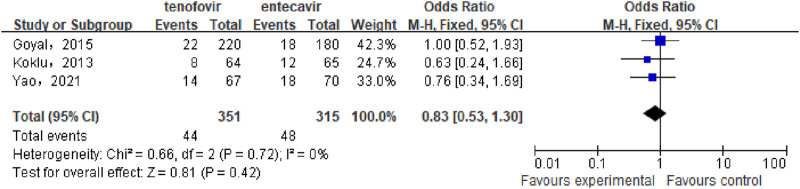
Incidence of Hepatic Encephalopathy in the tenofovir and entecavir Groups.

A total of 3 studies (Goyal et al., 2015; Köklü et al., 2013; Yao et al., 2021) involving 666 patients compared the incidence of hepatic encephalopathy between the tenofovir and entecavir groups in CHB-related cirrhosis patients. No statistical heterogeneity was observed among the included studies (P = 0.72, I^2^ = 0%), so a fixed-effect model was used for the analysis. The meta-analysis results showed that the incidence of hepatic encephalopathy in the tenofovir group (12.54%) was similar to that in the entecavir group (15.24%), with no statistically significant difference between the two groups [OR: 0.83, 95% CI: 0.53–1.30, P = 0.42], as shown in Figure 5.

#### 3.3.5 eGFR

**FIGURE 6:**
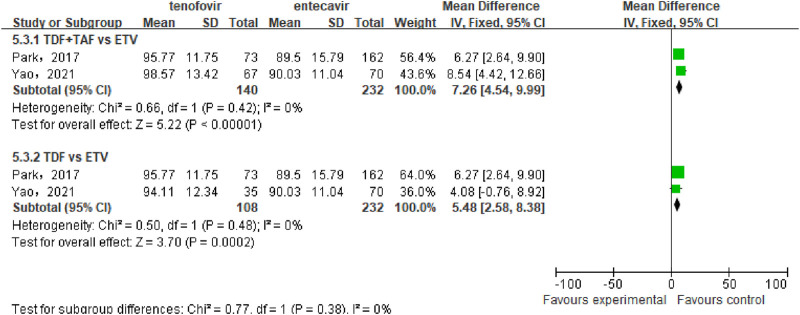
eGFR in the tenofovir and entecavir Groups after 48 weeks of Treatment.

A total of 2 studies (Park et al., 2017; Yao et al., 2021) involving 372 patients compared the eGFR between the tenofovir and entecavir groups after 48 weeks of treatment in CHB-related cirrhosis patients. No statistical heterogeneity was observed among the included studies (P = 0.42, I^2^ = 0%), so a a fixed-effect model was used for the analysis. The meta-analysis results indicated that the eGFR in the tenofovir group was significantly higher than that in the entecavir group after 48 weeks [WMD: 7.26, 95% CI: 4.54–9.99, P < 0.00001], as shown in Figure 6.

Subgroup analysis showed that TDF significantly increased the eGFR of patients compared to ETV [WMD: 5.48, 95% CI: 2.58–8.38, P = 0.0002]. Because only one article (Yao et al., 2021) compared the difference in eGFR between TAF and ETV, we did not conduct a combined analysis. However, the results of this article showed that the eGFR in the TAF group was significantly higher than that in the ETV group (103.44 ± 13.02 mL/min vs. 90.03 ± 11.04 mL/min) (Yao et al., 2021). Overall, compared to entecavir, tenofovir significantly increased the eGFR in CHB-related cirrhosis patients.

### 3.4 Publication bias

Egger’s test was conducted to evaluate the presence of publication bias in studies reporting cumulative HCC incidence and cumulative mortality. The results indicated no significant publication bias in the studies comparing tenofovir and entecavir for reducing cumulative HCC incidence (P = 0.568) and cumulative mortality (P = 0.095). Egger’s test was not performed for the other outcomes due to the small number of included studies.

### 3.5 Sensitivity analysis

**FIGURE 7:**
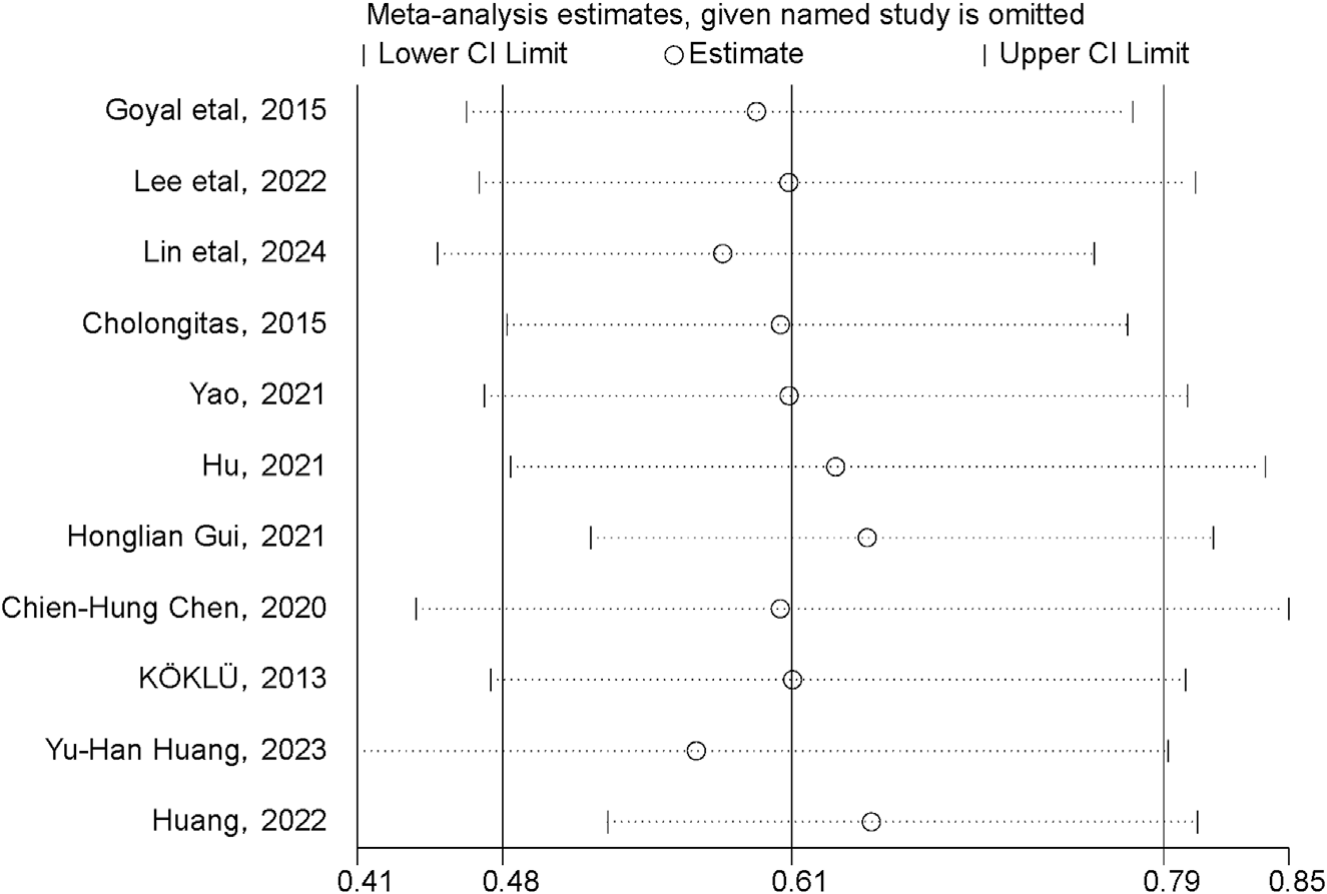
Sensitivity analysis of cumulative HCC incidence.

A sensitivity analysis was conducted to examine the influence of individual studies on the overall pooled effect. If the results remained unchanged after sensitivity analysis, it would suggest that the meta-analysis results were robust. If the sensitivity analysis revealed significant changes, it would indicate the presence of potential factors related to the intervention that could affect the credibility of the results. Sensitivity analysis was performed on the cumulative HCC incidence outcome (Figure 7), and the results showed no substantial changes in the pooled effect estimate, suggesting that the meta-analysis results were reliable.

## 4 Discussion

HBV infection is prevalent worldwide (You et al., 2023). According to the WHO, the global hepatitis B surface antigen (HBsAg) prevalence in the general population was 3.8% in 2019, with approximately 1.5 million new HBV infections, 296 million CHB patients, and 820,000 deaths from liver failure, cirrhosis, or HCC due to HBV infection. The intensity of HBV prevalence varies significantly across regions, influenced by factors such as the age of infection (You et al., 2023). China accounts for about one-third of the world’s HBV infections, with approximately 400,000 deaths annually from HBV-related complications, representing over 50% of global HBV deaths (Wen et al., 2020). HBV-related cirrhosis is a severe stage in the progression of hepatitis B and poses a significant threat to patients’ health and lives. Data from the 2019 Global Burden of Disease (GBD) study indicated that 331,000 people died from HBV-related cirrhosis and chronic liver disease in 2019, with a global age-standardized death rate (ASDR) of 4.03 per 100,000 population (Hsu et al., 2023), which did not show a significant decrease compared to the previous decade. In contrast, in China, the ASDR from cirrhosis and other chronic liver diseases caused by CHB was 6.69 per 100,000 (Cao G. et al., 2022). Thus, hepatitis B and HBV-related cirrhosis remain significant global public health challenges, particularly in high-prevalence countries like China, where further attention is needed.

For patients with HBV-related cirrhosis, whether in compensated or decompensated stages, antiviral therapy is recommended regardless of ALT and HBV-DNA levels or HBeAg status. Antiviral therapy is essential for improving liver function, slowing disease progression, and reducing HBV-related mortality. entecavir and tenofovir are currently recommended as first-line treatment options (You et al., 2023). tenofovir, including TDF and TAF, has shown strong antiviral efficacy, with long-term treatment significantly improving liver histology and maintaining a low resistance rate, with a cumulative 8-year resistance rate of nearly 0% (Liu et al., 2017). However, tenofovir may cause renal impairment, bone density loss, and lipid metabolism abnormalities (You et al., 2023), limiting its long-term use. entecavir is generally safer but carries a risk of resistance with long-term treatment. Studies have shown that the 5-year resistance rate for treatment-naive CHB patients on entecavir monotherapy is 1%–2% (Lee et al., 2014). Although some studies indicate that tenofovir may offer superior efficacy and safety compared to entecavir in treating CHB patients, high-quality evidence comparing the efficacy and safety of these two drugs in HBV-related cirrhosis patients requiring long-term antiviral therapy remains insufficient, warranting further exploration.

In this study, the cumulative incidence of HCC was significantly lower in HBV-related cirrhosis patients treated with tenofovir compared to those treated with entecavir in East Asian, suggesting that tenofovir significantly reduced the risk of HCC in these East Asian patients. This finding was consistent with several studies. For instance, Luo (2022) reported that tenofovir reduced the risk of HCC by more than threefold compared to entecavir in 213 CHB-related cirrhosis patients. Similarly, Huang et al. (2023) conducted a multicenter retrospective study in Taiwan involving 6,846 HBV-related cirrhosis patients and found that tenofovir reduced the incidence of HCC by approximately 40% compared to entecavir (Huang et al., 2023). A study involving 254 Japanese HBV patients showed that higher serum interferon-λ3 levels were detected in the patients treated with nucleotide analogues (adefovir or tenofovir) compared with those treated with nucleoside analogues (lamivudine or entecavir), Interferon-λ3 has demonstrated antitumor activity in some mouse cancer models, potentially explaining the difference in HCC risk between the two drugs (Murata et al., 2018). However, in non-East Asians, there was no significant difference in HCC incidence between the two groups, which was consistent with published research (Köklü et al., 2013). Additionally, the cumulative mortality in the tenofovir group was significantly lower than in the entecavir group in East Asian, consistent with findings from Huang et al., likely due to tenofovir’s ability to reduce the risk of cirrhosis-related complications (Huang et al., 2023). Although our study found no statistically significant differences in the incidence of hepatic encephalopathy between the two groups, the incidence was lower in the tenofovir group. In non East Asians, similar to the cumulative incidence of HCC, there was no significant difference in mortality between the two groups.

In terms of HBV-DNA clearance, our study showed no statistically significant differences between the tenofovir and entecavir groups at 24 and 48 weeks, consistent with several studies (Chen et al., 2020; Luo, 2022; Chen et al., 2018). For example, Chen et al. conducted a study involving 80 cirrhotic patients treated with either tenofovir or entecavir, and the results showed no significant differences in HBV-DNA clearance between the two groups at 48 weeks (Luo, 2022), indicating that both drugs improve liver histology in CHB-related cirrhosis patients with long-term treatment. Regarding renal function, eGFR changes were used as the assessment criteria, and the results showed that patients with HBV-related cirrhosis in the tenofovir group had higher eGFR levels at 48 weeks of treatment compared with patients in entecavir group, which was inconsistent with the reported higher risk of renal impairment with tenofovir compared with entecavir. We suspected that it was caused by the small number of included studies and the insufficient follow-up time, but such results at least indicated that tenofovir had little effect on renal function in patients within 1 year of use and did not lead to significant renal impairment.

This study has several strengths. First, to our knowledge, it is the first study to specifically evaluate the long-term efficacy and safety of tenofovir and entecavir in HBV-related cirrhosis patients. Second, this study included five clinical outcome measures, providing a comprehensive, multidimensional assessment of the advantages and disadvantages of these two antiviral drugs. Third, we conducted an extensive literature search, including more and newer studies than previous research. Finally, HBV infection and HBV-related cirrhosis represent a large population and a significant global public health challenge. tenofovir and entecavir are first-line treatment options, but there are few meta-analyses comparing their efficacy and safety in HBV-related cirrhosis, particularly in terms of cumulative HCC incidence and mortality, highlighting the importance of this study.

However, this study has some limitations. (1) Limitations in research design and data sources: The included studies were retrospective studies, which may have selection bias and recall bias, affecting the reliability of research results. (2) Most of the included studies are single center studies and only involve English literature, which may have some impact on the generalizability of the final conclusions. (3) Due to the limitation of the analysis content of the included studies, we did not analyze in further the efficacy and safety of tenofovir and entecavir in patients with chronic hepatitis B cirrhosis of different genders, and there are certain shortcomings. (4) Some studies had small sample sizes, which may limit the ability to assess the impact on clinical outcomes. (5) For some outcome measures, such as HBV-DNA clearance and eGFR, only a few studies were included, and there were no long-term follow-up results, which may introduce some bias. (6) The study combined patients with compensated and decompensated cirrhosis, which may limit the conclusions. (7) Because the number of studies included in the three outcome measures of HBV-DNA, hepatic encephalopathy, and eGFR was relatively small, correspondingly, the number of included races was also relatively small, which made it impossible for us to conduct further subgroup analysis to compare the differences between different ethnicities, resulting in certain limitations. (8) Due to the limited number of TAF-related studies, TAF and TDF were combined in this analysis, preventing an accurate evaluation of the individual efficacy and safety of TAF and TDF, which requires further investigation in future studies.

## 5 Conclusion

In summary, compared to entecavir, tenofovir significantly reduced the cumulative incidence of hepatocellular carcinoma and cumulative mortality in chronic hepatitis B-related cirrhosis in East Asian population. However, both drugs were comparable in terms of hepatitis B virus-deoxyribonucleic acid clearance and hepatic encephalopathy. tenofovir did not significantly cause renal dysfunction, but instead improved estimated glomerular filtration rate levels compared with entecavir. We look forward to prospective, large-scale randomized controlled trials to further confirm the efficacy and safety of these two drugs in treating HBV-related cirrhosis patients and to provide evidence for the optimal treatment strategies for this patient population.

## Data Availability

The original contributions presented in the study are included in the article/supplementary material, further inquiries can be directed to the corresponding authors.

## Abbreviations

HBV, Hepatitis B Virus; CHB, Chronic Hepatitis B; HCC, hepatocellular carcinoma; NA, Nucleos(t)ide analogues; ETV, entecavir; TDF, tenofovir disoproxil fumarate; TAF, tenofovir alafenamide fumarate; eGFR, estimated glomerular filtration rate; HBV-DNA, HBV-deoxyribonucleic acid; RCT, randomized controlled trial; NOS, Newcastle-Ottawa Scale; OR, odds ratio; CI, confidence interval; WMD, weighted mean difference; HBsAg, hepatitis B surface antigen; GBD, Global Burden of Disease; ASDR, age-standardized death rate.
